# Greater volumes of a callosal sub-region terminating in posterior language-related areas predict a stronger degree of language lateralization: A tractography study

**DOI:** 10.1371/journal.pone.0276721

**Published:** 2022-12-15

**Authors:** Victor Karpychev, Tatyana Bolgina, Svetlana Malytina, Victoria Zinchenko, Vadim Ushakov, Grigory Ignatyev, Olga Dragoy

**Affiliations:** 1 HSE University, Moscow, Russia; 2 Research and Practical Clinical Center for Diagnostics and Telemedicine Technologies of the Moscow Department of Health, Moscow, Russia; 3 National Research Center “Kurchatov Institute”, Moscow, Russia; 4 Institute for Advanced Brain Studies, Lomonosov Moscow State University, Moscow, Russia; 5 Institute of Linguistics, Russian Academy of Sciences, Moscow, Russia; University of Medicine & Dentistry of NJ - New Jersey Medical School, ISRAEL

## Abstract

Language lateralization is the most intriguing trait of functional asymmetry for cognitive functions. Nowadays, ontogenetic determinants of this trait are largely unknown, but there are efforts to find its anatomical correlates. In particular, a white matter interhemispheric connection–the corpus callosum–has been proposed as such. In the present study, we aimed to find the association between the degree of language lateralization and metrics of the callosal sub-regions. We applied a sentence completion fMRI task to measure the degree of language lateralization in a group of healthy participants balanced for handedness. We obtained the volumes and microstructural properties of callosal sub-regions with two tractography techniques, *diffusion tensor imaging (DTI)* and *constrained spherical deconvolution (CSD)*. The analysis of DTI-based metrics did not reveal any significant associations with language lateralization. In contrast, CSD-based analysis revealed that the volumes of a callosal sub-region terminating in the core posterior language-related areas predict a stronger degree of language lateralization. This finding supports the specific inhibitory model implemented through the callosal fibers projecting into the core posterior language-related areas in the degree of language lateralization, with no relevant contribution of other callosal sub-regions.

## Introduction

Functional asymmetry for some cognitive functions is one of the most prominent features of the brain [1]. Among other examples, there is a well-documented dominance of language in the left hemisphere in the majority of human beings [2, 3]. Only about 10–15% of individuals show atypical dominance of language processing in the right hemisphere or no clear hemispheric dominance, which is more frequently represented in left-handers and ambidexters than in right-handers [4–6]. Although language lateralization is associated with handedness, ontogenetic determinations of these traits differ [7]. While the causation of language lateralization remains largely unknown [8], efforts have been made to find its anatomical correlates in gray and white matter structures [9, 10].

Among gray matter structures, insular asymmetry has been suggested to predict language lateralization [11, 12]. However, although the insula is highly involved in different aspects of language processing, its role is not restricted to them [13], and as a consequence, an insular specific contribution to language lateralization is not still clear. In turn, asymmetry in clearly language-related areas, namely the planum temporale and Broca’s area, does not correlate with language lateralization [11, 14]. In contrast, interhemispheric white matter structures, mainly the corpus callosum (CC), which controls the functional interplay between hemispheres has been empirically associated with language lateralization [15]. Based on the fact of the less lateralized language functions in adults with agenesis of CC [16, 17], Adibpour, Dubois, Moutar, and Dehaene-Lambertz [18] explained it by the crucial role of the callosal fibers in infants with such disease. Therefore, the CC promotes the development of language lateralization in the early stages, and as a consequence, contributes to it in later life.

Two different models explain how the CC may contribute to language lateralization [19]. The excitatory model suggests the functional activation of both hemispheres through the CC because most of its fibers rely on excitatory glutamate neurotransmitters. The inhibitory model argues for the suppression of the subdominant hemisphere during language tasks by the dominant one through the inhibitory interneurons of the CC [20]. Depending on the different functions of the cortical areas involved in language processing, both excitation and inhibition might be ultimately carried out through the callosal sub-regions. Additionally, the functional diversity of the CC contributions might be even further enhanced by microstructural heterogeneity across callosal sub-regions [21]. Given that, the critical question is whether specific callosal sub-regions, rather than the CC as a whole, separately contribute to language lateralization and whether this contribution is excitatory or inhibitory [10].

Previous attempts to investigate the association between the CC and language lateralization used structural MRI and measured the midsagittal area of the CC based on it [10]. But firstly, structural MRI does not provide insight into microstructural properties, such as myelination, axonal diameter, and density of white matter fibers. Secondly, it does not properly assess individual variability in the overall shape of a tract [22] and, as a consequence, its volume, which is the closest proxy of the number of fibers [19]. This led to mixed results in previous structural MRI findings [22]. While Labache, Mazoyer, Joliot, Crivello, Hesling, and Tzourio-Mazoyer [23] and Bartha-Doering et al. [24] showed that a greater midsagittal area of the entire CC predicted decreased language lateralization, which was interpreted in favor of the excitatory model, Josse, Seghier, Kherif, and Price [25] showed the opposite effect and thus supported the inhibitory model.

In contrast to structural MRI, tractography allows researchers to reconstruct and quantify the volumes and microstructural properties of white matter tracts in a more direct way. Early tractography studies of language lateralization only investigated the microstructural properties obtained with *diffusion tensor imaging (DTI)*. Using the DTI metric of *fractional anisotropy (FA)*, Putnam, Wig, Grafton, Kelley, and Gazzaniga [26] and Steinman et al. [27] showed distinct contributions of the anterior and posterior callosal sub-regions to interhemispheric inhibition and excitation, respectively. However, both findings are inconsistent with Häberling, Badzakova-Trajkov, and Corballis [28], who revealed the excitation through the anterior callosal sub-region, and Westerhausen et al. [29], who, in turn, revealed the inhibition through the posterior callosal sub-region using another DTI metric, *relative anisotropy*. Of note, none of these studies analyzed the volumes of the callosal sub-regions. Thus, the application of the DTI technique did not result in consistent evidence about the contribution of callosal sub-regions to interhemispheric regulation in language processing. This may be because DTI cannot reliably quantify structural properties because of its poor ability to resolve multiple crossings of fibers in the CC [30]. Thus, DTI does not fully reconstruct all callosal fibers [31] and underestimates the volumes of callosal sub-regions. Likewise, DTI represents the microstructural properties of underlying tissue only to a limited extent [32, 33]. For example, the most frequently used DTI metric, FA, is believed to be sensitive to fiber density [34, 35] and myelin content of fibers [36]. But Friedrich et al. [37] demonstrated that FA does not coincide with fiber density in the posterior callosal sub-region and myelin content of fibers in the most callosal sub-regions.

To overcome the limitations of DTI, a more advanced tractography technique, *constrained spherical deconvolution (CSD)*, is more appropriate for modeling the crossing fibers [38], as the latter allows a more precise evaluation of the volumes of callosal sub-regions [31]. But, as with DTI, CSD has not been used to investigate the association between volumes of the callosal sub-regions and language lateralization. To fill this gap, we applied both tractography techniques in the present study and tested the extent of the DTI limitations in comparison to CSD. In addition to extracting volumes using the two approaches, we followed previous DTI studies and examined the microstructural properties of the callosal fibers across sub-regions and their association with language lateralization. For that, we used FA in DTI [37] and *hindrance modulated orientational anisotropy (HMOA)* in CSD, which reflects axonal diameter, fiber density and dispersion and represents the microstructural properties of fibers more precisely than FA [39]. HMOA was previously used in association with spatial attention lateralization [40] but has not yet been applied to study language lateralization.

Finally, in previous DTI studies aiming to reveal the contribution of the CC to language lateralization, the latter was measured with fMRI paradigms using word generation [26, 28, 29] or listening tasks [27]. These two tasks mainly activate the anterior or posterior language-related areas, respectively. Thus, each previous DTI study reported a result that was based on language lateralization in either the anterior or posterior language-related areas, but critically, not in both. Thus, distinctions in the associations of the structural properties of callosal sub-regions and language lateralization in the anterior or posterior language-related areas have been based on studies with different groups of participants, but not within the same group using the same task. In the present study, we measured language lateralization using a more comprehensive sentence completion task with fMRI that robustly activates the anterior and posterior language-related areas [41–43] within the same individual. To ensure variability of the degree of language lateralization [1], we balanced our participants on handedness and recruited right-handed, left-handed, and ambidextrous participants.

Overall, the goal of the study was to measure both the volumes and microstructural metrics of callosal sub-regions using DTI and CSD, and to test their specific associations with the degree of language lateralization, as derived from a comprehensive fMRI task in a cohort of participants ensuring the variability of such asymmetry.

## Materials and methods

### Participants

Fifty neurologically healthy individuals participated in the study (32 females; mean age = 24.4, *SD* = 4.8, range 18–37 years). All participants were Russian native speakers with no history of psychiatric or neurological diseases. The handedness of each participant was estimated with ten questions of the Edinburgh inventory [44]. In our study, 20 participants with scores from +45 to +100 were right-handed (13 females; mean age = 24.9, *SD* = 5.7, range = 18–37 years), 20 participants with scores from -100 to -45 were left-handed (14 females; mean age = 23.8, *SD* = 4.0, range = 19–30 years), and ten participants with scores from -45 to +45 were ambidextrous (7 females; mean age = 23.2, *SD* = 3.1, range = 19–27 years). The study was conducted in accordance with the Declaration of Helsinki, all participants gave written informed consent, and the protocol was approved by the local ethical committee of the National Research Center “Kurchatov Institute” (Moscow, Russia).

### Language task during fMRI

All participants performed a block-designed language task with alternating sentence completion and baseline blocks, lasting 21.3 s each. Each block consisted of three stimuli presented for 5 s, which were separated by an inter-stimulus interval with an exclamation mark on the screen presented for 2.1 s. In each trial of a sentence completion block, participants had to read aloud a visually presented Russian sentence and complete it with a semantically and grammatically appropriate final word (e.g., *Teper’ ministr podpisyvaet vazhnoe… Now the minister in signing an important…*). All sentences consisted of an adverb of time, a subject, and a predicate with an omitted direct object. One half of the sentences were expanded with an adjective before the object. The other half of the sentences were expanded with an adverb before the predicate. In each trial of the baseline block, participants had to read aloud a string of four syllables and repeat the same syllable one more time (e.g., *Peeeee peeeeeeeeee peeeeeee peeeeeee…*). The length of the strings of syllables, measured in letters and syllables, was matched to the length of the sentences in sentence completion blocks. The scanning session consisted of two runs, and each run included 120 trials (60 sentence completion and 60 baseline trials), lasting 14 min and 37 s. All participant responses were audio recorded.

### MRI acquisition

We performed MRI data acquisition on a Siemens 3T Magnetom Vario MRI scanner. Functional T2* images were acquired using an EPI sequence with TR = 7000 ms; TE = 30 ms; flip angle = 90°. Each functional T2* image contained 40 axial slices (no gap) with FOV = 205×205 mm^2^, spatial resolution = 3×3×3 mm^3^. An additional field map for fMRI data was acquired using a dual-echo GRE sequence with TR = 400 ms; TE_1_ = 4.92 ms; TE_2_ = 7.38 ms; 30 slices (no gap); FOV = 205×205 mm^2^; spatial resolution = 3×3×3 mm^3^. Sparse sampling acquisition was used to record the participant’s overt responses in intervals that were equal to TR delay = 5 s. Structural T1 images were obtained using a 3D MP-RAGE sequence with TR = 1900 ms; TE = 2,2 ms; flip angle = 9°. Each T1-image contained 176 axial slices (no gap) with FOV = 320×320 mm^2^ and spatial resolution = 1×1×1 mm^3^. Diffusion-weighted images (DWIs) were acquired using single-shot spin echo EPI sequence with TR = 13700 ms; TE = 101ms; 65 axial slices (no gap); FOV = 240×240 mm^2^; spatial resolution = 2×2×2 mm^3^; b-value = 1500 s/mm^2^. Sixty-four diffusion directions and one image with b = 0 s/mm^2^ were collected in AP phase encoding direction, twice for 47 participants and once for 3 participants. An additional field map for the DWIs was acquired using a dual-echo GRE sequence with TR = 698 ms; TE_1_ = 4.92 ms; TE_2_ = 7.38 ms; 65 slices (no gap); FOV = 240×240 mm^2^; spatial resolution = 2×2×2 mm^3^.

### DWI analysis

We pre-processed the DWI data in FMRIB Software Library (FSL) (https://fsl.fmrib.ox.ac.uk/fsl/fslwiki/FSL). No DWI data were removed after visual estimation of quality. For 47 participants, two diffusion-weighted sequences in the AP phase encoding direction were merged to improve the signal-to-noise ratio. For all participants, DWIs were corrected for the eddy-current and subject motion distortions by aligning to the images with b = 0 s/mm^2^, and for the EPI distortion by applying the field map.

For all participants, the DTI technique was applied in ExploreDTI software (http://www.exploredti.com); the CSD technique was applied in StarTrack software (https://www.mr-startrack.com). For DTI, the diffusion tensor was fitted at each voxel using the linear approach. For CSD, the damped Richardson-Lucy algorithm was applied with fiber response = 1.5×10^−3^ mm^2^/s^−1^, 400 iterations, relative threshold = 2×10^−3^, and geometric damping parameter = 16 [45] to obtain the fiber orientation distribution at each voxel. Both whole-brain tractographies were performed using the maximum angle = 30°, seed point resolution = 1 mm^3^, and step size = 1 mm. FA threshold = 0.2 was used for DTI; absolute threshold = 2×10^−3^ was used for CSD. In addition, FA in DTI and HMOA in CSD maps were obtained in the native space of each participant.

**Fig 1:**
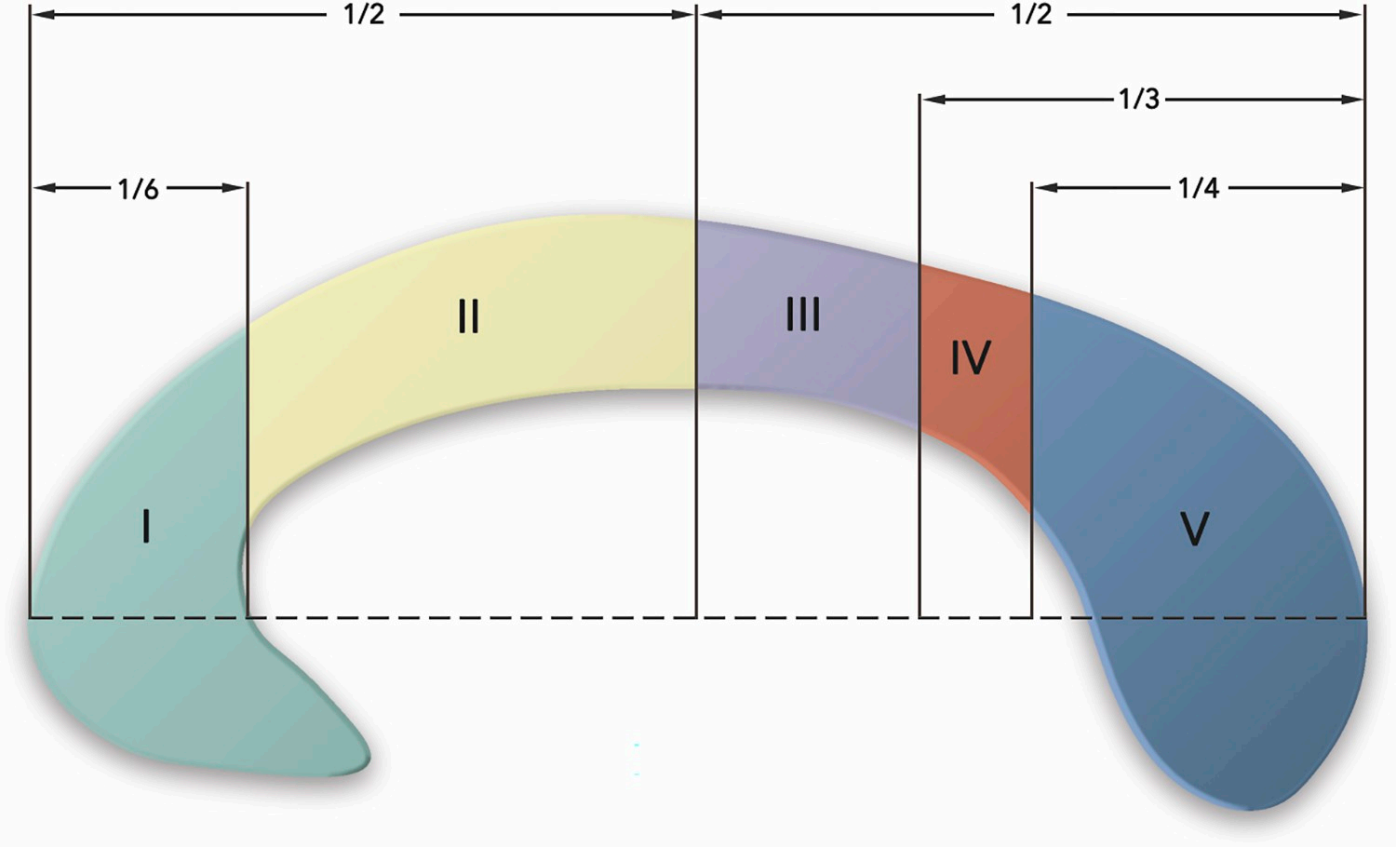
Hofer’s scheme of callosal sub-regions. The image was adapted from [46].

**Fig 2:**
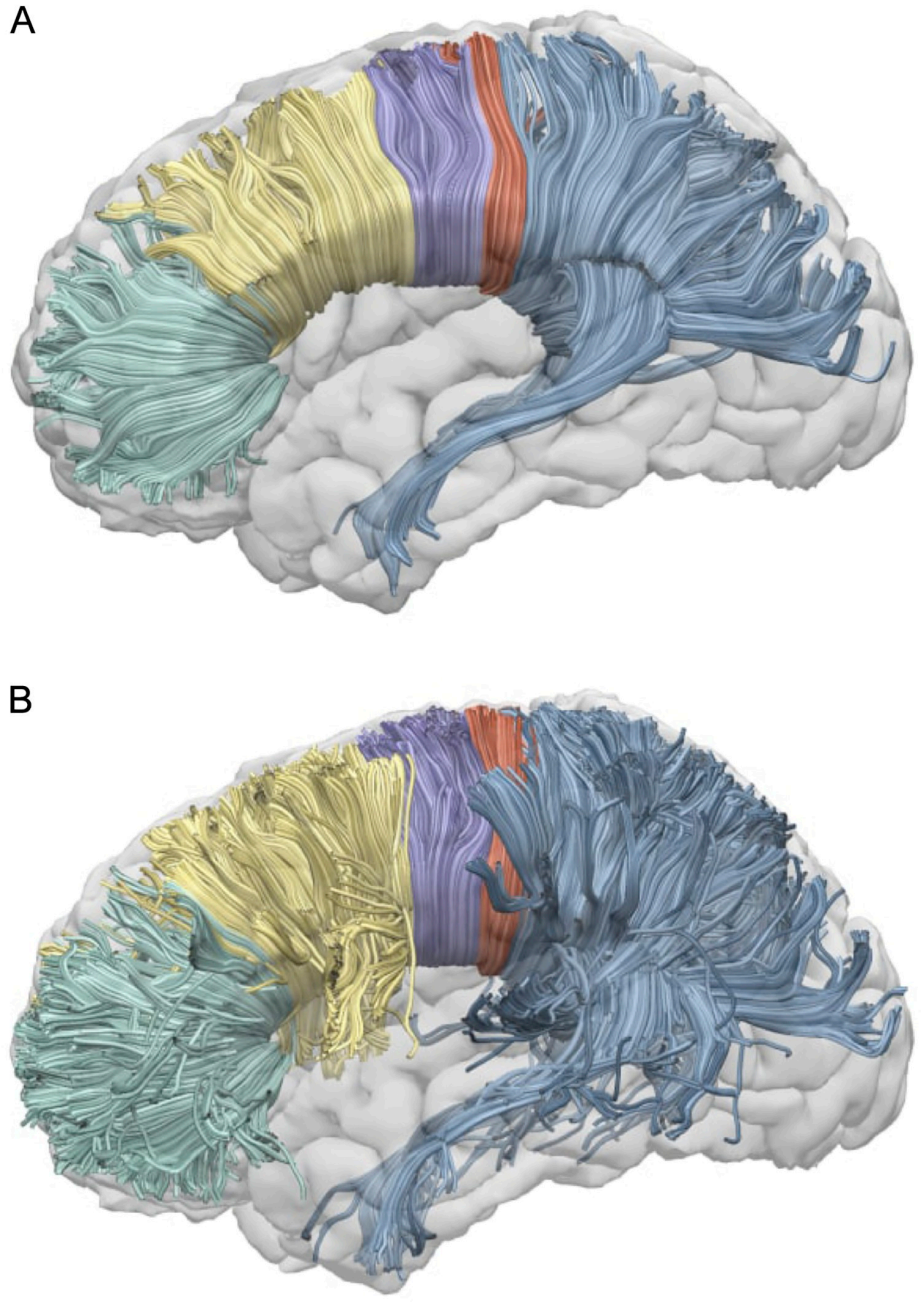
Single subject reconstruction of callosal sub-regions. The visualization of a single subject reconstruction of callosal sub-regions according to Hofer’s scheme in DTI (A) and CSD (B). DTI = diffusion tensor imaging; CSD = constrained spherical deconvolution.

The CC was manually reconstructed from the whole-brain tractographies in native space of each participant in TrackVis software (http://trackvis.org). For both whole-brain tractographies of each participant, an inclusion region of interest (ROI) was drawn manually in the midsagittal slice of the CC on the FA map and divided into sub-regions according to Hofer’s scheme [46]. In contrast to other schemes, Hofer’s scheme is based on tractography results. Fig 1 presents the Hofer’s scheme of the CC. In addition, exclusion ROIs were manually placed for each participant to remove spurious fibers. The reconstructed callosal sub-regions in both DTI and CSD corresponded to CC-I, with fibers projecting into the prefrontal cortex (PFC); CC-II—premotor cortex and supplementary motor area (PM-SMA); CC-III—primary motor cortex (M1); CC-IV—primary somatosensory cortex (S1); CC-V—parietal, temporal, occipital lobes (PTOLs) in Hofer’s scheme [46]. Fig 2 presents the visualization of a single subject reconstruction of callosal sub-regions performed in Surf Ice (https://www.nitrc.org/projects/surfice/).

The volumes in DTI and CSD of each sub-region were extracted in TrackVis and normalized by the total volume of gray and white matter obtained from the T1 image of each participant. The normalized volume of a sub-region is further referred to as volume for simplicity. Also, for each sub-region, values of FA and HMOA across streamlines were extracted from the corresponding maps in TrackVis.

### fMRI analysis

We analyzed fMRI data with SPM12 (https://www.fil.ion.ucl.ac.uk/spm/software/spm12/) in MATLAB R2017b (MathWorks; Natick, MA, USA). The first four volumes of each run corresponding to the task instructions were discarded. For preprocessing, voxel displacement maps were estimated based on the acquired field maps. Functional images were manually aligned along the anterior commissure-posterior commissure line. Using the voxel displacement maps, functional images were simultaneously unwarped to correct for magnetic field inhomogeneity and re-aligned to correct for head motion. The structural T1 image was co-registered to the mean functional volume. The structural T1 and functional T2* images were normalized into standardized stereotactic space (MNI 152-subject template) based on segmentation into gray matter, white matter, and cerebrospinal fluid. The functional T2* images were spatially smoothed with an 8-mm FWHM isotropic Gaussian kernel.

**Fig 3:**
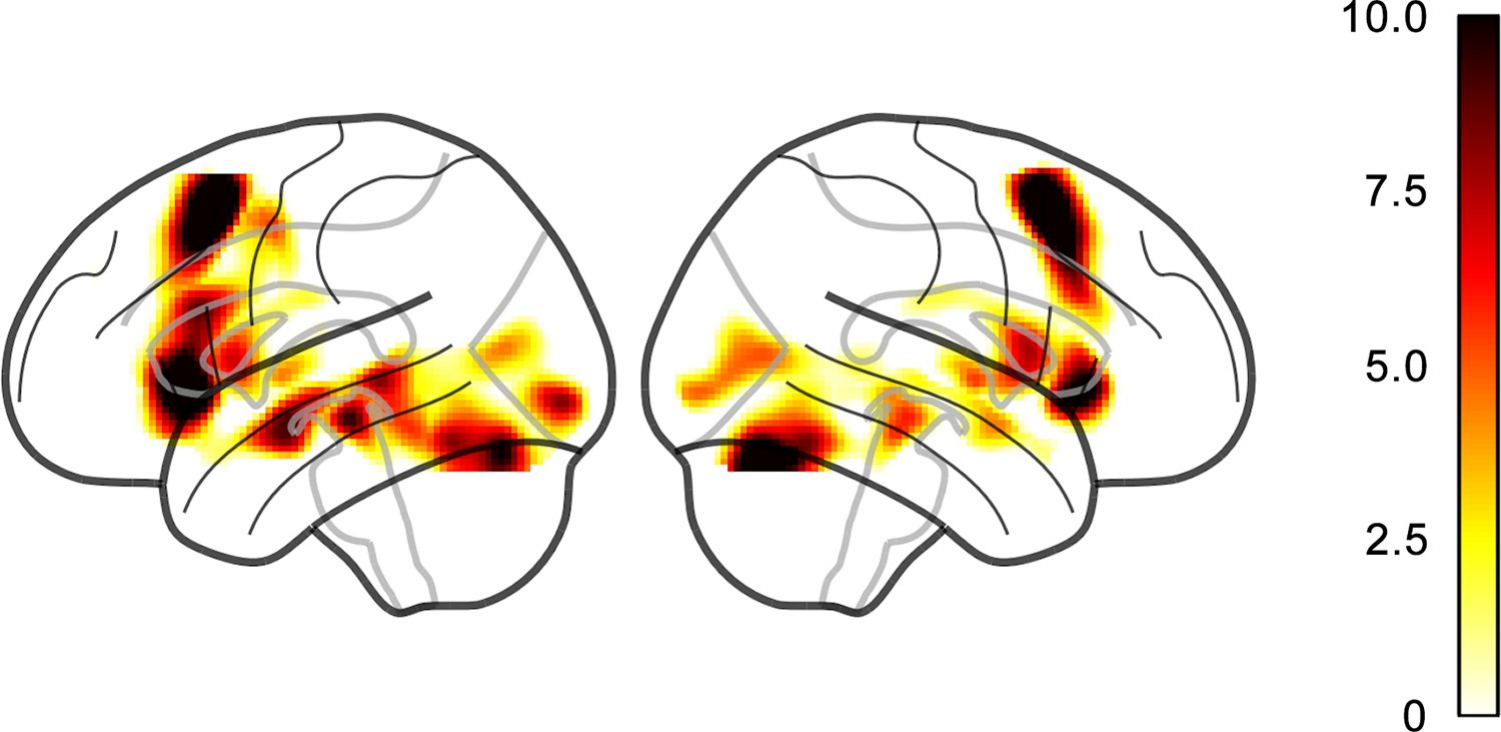
Group map of language activation. Colors indicate statistically significant language activation (family-wise error rate correction, *p* < 0.05): *sentence completion block* > *baseline block*.

After preprocessing, we conducted the first-level statistical analysis to obtain an individual map of language-related activation for each participant. The two conditions (sentence completion and baseline blocks) were modeled in an event-related design where each stimulus corresponded to an event of 7.1 s duration. The model also included regressors: six motion parameters obtained in realignment, as well as binary response accuracy scored independently by two raters. A canonical hemodynamic response function with no derivatives was used to model BOLD response. A high pass filter of 256 s was used to eliminate low-frequency scanner drift and a first-order autoregressive model was used to correct for autocorrelations. A group map of language activation was obtained by contrasting the sentence completion to the baseline condition with a one-sample *t*-test in Python, version 3.7 (https://www.python.org) using the “*nilearn*” package (https://nilearn.github.io). Results were considered significant at *p* < 0.05 with family-wise error rate correction for multiple comparisons. Fig 3 shows the group map of language activation.

The language lateralization index (LI) was calculated for multiple regions for each participant using the fMRI activation maps obtained in the first-level statistical analysis in the LI-toolbox [47] in SPM12 according to the formula:

$$LI=[\left(A_{L}-A_{R})/(A_{L}+A_{R}\right)]$$

whereby A_L_ and A_R_ are language-related activation of a region in the left and right hemispheres, respectively. For each participant, the fMRI activation map was divided into cortical areas–PFC, PM-SMA, M1, S1, and PTOLs–obtained in the Brainnetome atlas [48] according to the callosal sub-regions [46]. Table 1 presents the correspondence between the callosal sub-regions and selected ROIs. The PM-SMA included the core language-related areas, such as the pars triangularis, pars opercularis [49], and supplementary motor area [50], whereas the PTOLs included the angular and supramarginal gyri, and posterior temporal cortex, which are also frequently associated with language processing [49]. In contrast, the PFC, M1, and S1 did not include core language-related areas, instead being associated with executive functions, general motor, and somatosensory processing, respectively [51–53]. For each of these ROIs, LI calculations were based on voxel count and voxel values, using adaptive thresholding as implemented in the LI-toolbox and excluding 5 mm along the brain midline. The LI could range from -1 for strong right-hemispheric lateralization to +1 for strong left-hemispheric lateralization. Following [54], our study used absolute values of the resulting LI, so that stronger lateralization, regardless of the hemisphere, corresponded to values closer to +1, whereas weaker lateralization corresponded to values closer to 0. The absolute values indicating the degree of lateralization are further referred to as LI_abs_ for simplicity. Regarding the hemispheric dominance, we, additionally, considered raw values of resulting LI, which are further referred to as LI_raw_ for simplicity.

**Table 1:** Correspondence between the callosal sub-regions and cortical areas.

Callosal sub-region	CC-I	CC-II	CC-III	CC-IV	CC-V
Cortical area	PFC	PM-SMA	M1	S1	PTOLs

CC = corpus callosum; PFC = prefrontal cortex; PM-SMA = premotor cortex and supplementary motor area; M1 = primary motor cortex; S1 = primary somatosensory cortex; PTOLs = parietal, temporal, and occipital lobes.

### Statistical analyses

All statistical analyses were performed in JASP (https://jasp-stats.org) and RStudio, version 4.2.0 (https://www.rstudio.com) using package “*BayesFactor*” (https://github.com/richarddmorey/BayesFactor). Following recommendations [55], we reported the results of each analysis in both frequentist statistics and Bayesian statistics via Bayes factors (*BF*_*10*_). *BF*_*10*_ is a change from an odds ratio between prior probabilities of the alternative and null hypotheses to an odds ratio of their posterior probabilities driven by the observed data. While the frequentist statistics only either reject or not the null hypothesis, *BF*_*10*_ allows us to quantify evidence in favor of either the null hypothesis (*BF*_*10*_ < 1/3) or the alternative hypothesis (*BF*_*10*_ > 3) or, as an additional state, indicate no clear evidence in favor of either hypothesis (1/3 < *BF*_*10*_ < 3) [55, 56]. We calculated *BF*_*10*_ using a default prior probability distribution, a Cauchy distribution with a center location, *x*_*0*_  =  0; scale value, *σ * =  √2/4 [57]. For analyses with multiple testing, we adjusted this distribution by decreasing a scale value proportionally to the number of tests [58, 59]. Thus, an effect of either difference between groups or predictors of multiple regressions became closer to 0. Details of the adjustment procedure are presented in [60].

To test whether CSD provided greater volume than DTI, the difference in the volumes of each callosal sub-region between DTI and CSD was assessed with a paired samples *t*-test. Due to multiple testing in this analysis, we adjusted the level of significance and scale value for five tests (Bonferroni correction, *α* = .05/5 = .01; *σ * = 0.072). To compare the microstructural heterogeneity of the callosal fibers across all callosal sub-regions, one-way ANOVA tests were used separately for FA in DTI, and for HMOA in CSD (Bonferroni correction, *α* = .05/2 = .025; *σ* = 0.18). To assess the association of the structural properties of callosal sub-regions with LI_abs_, we used general multiple regression. For each pair of the callosal sub-region and respective LI_abs_ in that region, two general multiple regressions using backward elimination were built with either the volume and FA in DTI, or the volume and HMOA in CSD, as independent variables. To obtain *BF*_*10*_ for the general multiple regressions, we additionally built Bayesian multiple regressions. *BF*_*10*_ were shown for the three possible models: a model with both independent variables, and two models with one of the two independent variables, compared to the null model without any independent variables [61]. For each of these three models and a null model, a prior probability of 0.25 was defined. Due to multiple testing of LI_abs_ in this analysis across all callosal sub-regions in DTI and CSD, we adjusted the level of significance for (Bonferroni correction, *α* = .05/10 = .005) and scale value (*σ* = 0.036) for ten general and Bayesian multiple regressions.

### Supplementary analyses

As a supplementary analysis, we explored the associations between the structural properties of the callosal sub-regions and LI_raw,_ thus regarding the hemisphere. Due to multiple testing of LI_raw_ in this analysis across all callosal sub-regions in DTI and CSD, we adjusted the level of significance (Bonferroni correction, *α* = .05/10 = .005) and scale value (*σ* = 0.036) for ten general and Bayesian multiple regressions.

Finally, to test whether obtained associations of the structural properties of callosal sub-regions with LI_abs_ and LI_raw_ depend on handedness, we conducted an analysis dividing all participants according to their handedness. We distinguished two groups, which corresponded to *typical handedness (TH)* and *atypical handedness (AH)* and consisted of right-handers and left-handers with ambidexters, respectively. For each pair of the callosal sub-region and respective either LI_abs_ or LI_raw_, we built general multiple regressions with the structural properties in both DTI and CSD, as independent variables, additionally nested by the groups of handedness. *BF*_*10*_ were shown for these models based on comparing to a null model. Due to multiple testing of LI_abs_ and LI_raw_ in this analysis, we adjusted the level of significance (Bonferroni correction, *α* = .05/20 = .0025) and a scale value (*σ* = 0.018) for 20 general and Bayesian multiple regressions.

## Results

### Functional lateralization

Table 2 presents the LI_raw_ and LI_abs_ of the cortical areas corresponding to the callosal sub-regions. Detailed descriptive statistics of the LI_raw_ and LI_abs_ according to handedness of the participants are presented in the (S1 Table).

**Table 2:** Descriptive statistics of the LI_raw_ and LI_abs_ in the selected ROIs.

	PFC		PM-SMA		M1		S1		PTOLs	
	LI_raw_	LI_abs_	LI_raw_	LI_abs_	LI_raw_	LI_abs_	LI_raw_	LI_abs_	LI_raw_	LI_abs_
Mean	0.29	0.47	0.41	0.64	0.12	0.34	0	0.33	0.29	0.37
*SD*	0.44	0.23	0.56	0.27	0.44	0.30	0.43	0.26	0.32	0.21
Min	-1.0	0.06	-0.99	0.01	-0.99	0	-0.90	0	-0.48	0
Max	0.85	1.0	0.99	0.99	1.0	1.0	0.98	0.98	0.84	0.84

PFC = prefrontal cortex; PM-SMA = premotor cortex and supplementary motor area; M1 = primary motor cortex; S1 = primary somatosensory cortex; PTOLs = parietal, temporal, and occipital lobes; SD = standard deviation.

Left-hemispheric language lateralization was found across 40 in the PFC (12 left-, 19 right-handers, and 9 ambidexters), 39 in the PM-SMA (11 left-, 19 right-handers, and 9 ambidexters), 35 in the M1 (12 left-, 14 right-handers, and 9 ambidexters), 25 in the S1 (10 left-, 10 right-handers, and 5 ambidexters), and 39 in the PTOLs (12 left-, 18 right-handers and 9 ambidexters) from 50 participants.

### Callosal metrics

For all callosal sub-regions, volumes were significantly greater in CSD than in DTI, with evidence in favor of the significant differences (*BF*_*10*_ > 10^5^; Table 3).

**Table 3:** Results of a paired samples *t*-test comparing the volumes of each callosal sub-region between DTI and CSD.

**Callosal sub-region**	**Volume in DTI**		**Volume in CSD**		*t*(49)	*p*	*BF*_*10*_
	*M*	*SD*	*M*	*SD*			
CC-I	16.1	2.7	33.7	9.2	14.36	< 0.001	> 10^5^
CC-II	18.8	3.4	44.5	12.8	16.09	< 0.001	> 10^5^
CC-III	9.9	2.2	18.0	5.8	12.26	< 0.001	> 10^5^
CC-IV	8.5	2.0	14.8	7.0	7.10	< 0.001	> 10^5^
CC-V	35.3	6.3	67.8	19.8	12.40	< 0.001	> 10^5^

Both volumes in DTI and CSD are multiplied by 10^−3^. CC = corpus callosum; DTI = diffusion tensor imaging; CSD = constrained spherical deconvolution; M = mean; SD = standard deviation; BF_10_ = Bayes factors.

**Fig 4:**
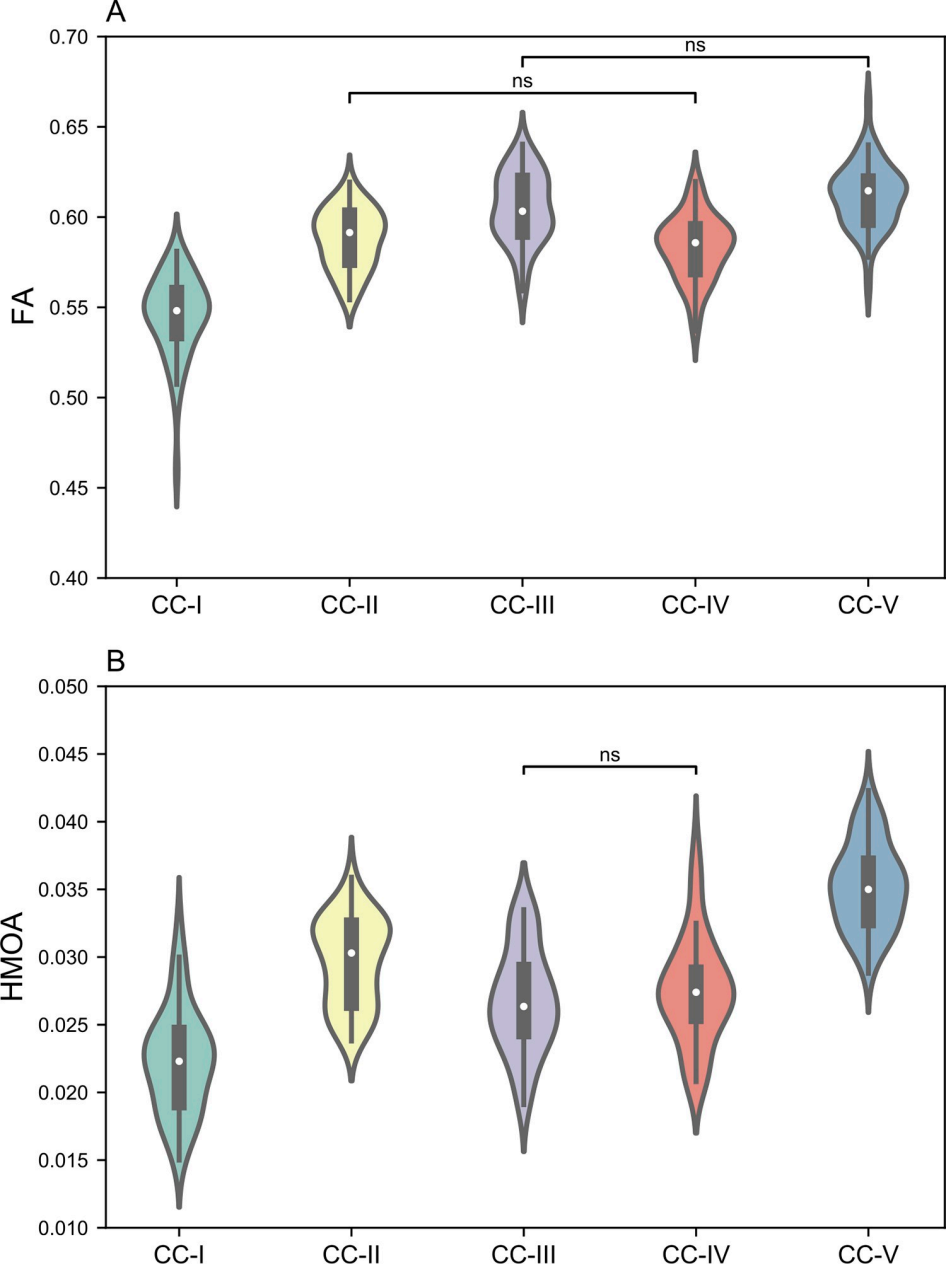
FA and HMOA across all callosal sub-regions. The sub-regions significantly differed in FA (A) except for the comparisons between CC-II and CC-IV, as well as between CC-III and CC-V. The sub-regions significantly differed in HMOA (B) except for the comparisons between CC-III and CC-IV. CC = corpus callosum; FA = fractional anisotropy; HMOA = hindrance modulated orientational anisotropy; ns = non-significant.

The one-way ANOVA tests revealed significant differences across callosal sub-regions in FA in DTI (*F*_(4,245)_ = 94,38, *p* < 0.001) and in HMOA in CSD (*F*_(4,245)_ = 86,41, *p* < 0.001). *BF*_*10*_ > 10^4^ for both tests indicated evidence for these differences. *Post hoc* two-sample *t*-tests (Bonferroni correction, *α* = .05/10 = .005) showed that all callosal sub-regions significantly differed in FA, except for the comparisons between CC-II and CC-IV (*t*_(49)_ = 2.12, *p* = 0.04) and between CC-III and CC-V (*t*_(49)_ = -2.43, *p* = 0.02). *BF*_*10*_ = 1.40 and *BF*_*10*_ = 1.75 (*σ* = 0.036) for the comparisons between CC-II and CC-IV, and between CC-III and CC-V, respectively, showed no clear evidence in favor of difference. *Post hoc* two-sample *t*-tests (Bonferroni correction, *α* = .05/10 = .005) also showed that all callosal sub-regions significantly differed in HMOA, except for the comparison between CC-III and CC-IV (*t*_(49)_ = -1.84, *p* = .071). *BF*_*10*_ = 0.74 (*σ* = 0.036) for this comparison showed no clear evidence in favor of no difference. Fig 4 presents FA and HMOA across all callosal sub-regions. Detailed statistics of the callosal metrics according to handedness of the participants are presented in the (S2 Table).

### Relations of the LI_abs_ to the structural properties of callosal sub-regions

Table 4 presents the results of the general linear regressions of LI_abs_. The LI_abs_ in the cortical areas corresponding to the callosal sub-regions were not significantly related to DTI-based volumes nor to FA in these sub-regions. There was an association between stronger LI_abs_ in the PTOLs and greater FA of CC-V (*β* = 3.65, *SE* = 1.67, *t*_(48)_ = 2.18, *p* = 0.03), which did not reach Bonferroni corrected significance level (*α* = .005). Bayesian multiple regression showed no clear evidence for this relation (*BF*_*10*_ = 1.46).

**Table 4:** Results of the general multiple regressions in DTI and CSD examining the relations of LI_abs_ to volumes and FA in DTI, and to volumes and HMOA in CSD.

	**Global model**				**Selected model**			
	*β*	*SE*	*t*(47)	*p*	*β*	*SE*	*t*(48)	*p*
**CC-I**								
**DTI**								
(Intercept)	-0.08	0.82	-0.10	0.92	-0.20	0.79	-0.25	0.80
Volume	-7.00	11.90	-0.59	0.56	NA	NA	NA	NA
FA	1.22	1.46	0.84	0.41	1.22	1.45	0.85	0.40
**CSD**								
(Intercept)	0.23	0.24	0.95	0.35	0.28	0.12	2.30	0.03
Volume	5.88	3.53	1.67	0.10	5.75	3.44	1.67	0.10
HMOA	1.85	8.58	0.22	0.83	NA	NA	NA	NA
**CC-II**								
**DTI**								
(Intercept)	0.79	1.38	0.57	0.57	0.36	0.19	1.92	0.06
Volume	0.01	0.01	1.48	0.145	0.01	0.01	1.46	0.15
FA	-0.74	2.38	-0.31	0.76	NA	NA	NA	NA
**CSD**								
(Intercept)	0.61	0.37	1.65	0.11	0.31	0.13	2.37	0.02
Volume	7.23	2.82	2.56	0.01	7.40	2.81	2.64	0.01
HMOA	-9.88	11.34	-0.87	0.39	NA	NA	NA	NA
**CC-III**								
**DTI**								
(Intercept)	0.44	1.42	0.31	0.76	0.42	0.19	2.14	0.04
Volume	-8.04	19.57	-0.41	0.68	-7.99	19.15	-0.42	0.68
FA	-0.038	2.28	-0.017	0.99	NA	NA	NA	NA
**CSD**								
(Intercept)	0.67	0.36	1.86	0.07	0.72	0.30	2.41	0.02
Volume	2.30	7.40	0.21	0.76	NA	NA	NA	NA
HMOA	-13.83	11.44	-1.21	0.23	-14.46	11.16	-1.30	0.20
**CC-IV**								
**DTI**								
(Intercept)	0.51	1.24	0.41	0.68	0.19	0.16	1.98	0.24
Volume	17.95	19.37	0.93	0.36	16.62	18.50	0.90	0.37
FA	-0.57	2.19	-0.26	0.80	NA	NA	NA	NA
**CSD**								
(Intercept)	0.29	0.28	1.03	0.31	0.27	0.09	3.12	0.003*
Volume	4.47	5.32	0.84	0.41	4.47	5.26	0.85	0.40
HMOA	-0.67	9.55	-0.07	0.94	NA	NA	NA	NA
**CC-V**								
**DTI**								
(Intercept)	-1.79	1.02	-1.76	0.09	-1.86	1.03	-1.82	0.08
Volume	6.07	4.72	1.29	0.21	NA	NA	NA	NA
FA	3.18	1.70	1.87	0.07	3.65	1.68	2.18	0.03
**CSD**								
(Intercept)	-0.04	0.34	-0.10	0.92	0.07	0.10	0.69	0.49
Volume	4.49	1.42	3.13	0.003*	4.45	1.41	3.15	0.003*
HMOA	2.89	9.05	0.32	0.75	NA	NA	NA	NA

Global model with volume and FA in DTI, and volume and HMOA in CSD as independent variables; model selected by backward elimination with either volume or FA in DTI, either volume or HMOA in CSD. CC = corpus callosum; FA = fractional anisotropy; DTI = diffusion tensor imaging; HMOA = hindrance modulated orientational anisotropy; CSD = constrained spherical deconvolution; SE = standard error; NA = not applicable.

*Predictors significant at *α* = .005 Bonferroni corrected.

There was an association of stronger LI_abs_ in the PM-SMA and greater CSD-based volume of CC-II (*β* = 7.40, *SE* = 2.81, *t*_(48)_ = 2.64, *p* = 0.01), which did not reach *α* = .005. Bayesian multiple regression showed no clear evidence for this relation (*BF*_*10*_ = 2.12). We also found a significant association between stronger LI_abs_ in the PTOLs and greater CSD-based volume of CC-V (*β* = 4.45, *SE* = 1.41, *t*_(48)_ = 3.15, *p* = 0.003). Bayesian multiple regression showed evidence for this relation (*BF*_*10*_ = 4.00). Relations of the other pairs of LI_abs_ in the cortical areas to either CSD-based volumes or HMOA of any sub-regions were not significant. Detailed summaries of the results of Bayesian multiple regressions are presented in the (S3 Table).

### Supplementary analyses

#### Relations of the LI_raw_ to the structural properties of callosal sub-regions

The supplementary analysis indicated a significant association between stronger LI_raw_ in the PFC and greater CSD-based volume of CC-I (*β* = 19.65, *SE* = 6.33, *t*_(48)_ = 3.10, *p* = 0.003). However, Bayesian multiple regression showed no clear evidence for this relation (*BF*_*10*_ = 2.97). There were associations of stronger LI_raw_ in the PM-SMA and greater CSD-based volume of CC-II (*β* = 13.64, *SE* = 5.83, *t*_(47)_ = 2.31, *p* = 0.03), and stronger LI_raw_ in the PTOLs and greater CSD-based volume of CC-V (*β* = 5.35, *SE* = 2.16, *t*_(47)_ = 2.56, *p* = 0.01), which did not reach *α* = .005. Bayesian multiple regression showed no clear evidence for these relations *BF*_*10*_ = 1.85 and *BF*_*10*_ = 1.91, respectively. Detailed summaries of the results of the general and Bayesian multiple regressions are presented in the (S4 and S5 Tables).

#### Analysis of the relations within handedness groups

*LI*_*abs*_. We found a significant association for AH between stronger LI_abs_ in PM-SMA and CSD-based volume of CC-II (*β* = 12.24, *SE* = 3.55, *t*_(47)_ = 3.44, *p* = 0.001). But Bayesian multiple regression showed no clear evidence for this relation for AH (*BF*_*10*_ = 1.62). Also, for AH, there was an association between stronger LI_abs_ in the PTOLs and CSD-based volume of CC-V (*β* = 5.23, *SE* = 2.0, *t*_(47)_ = 2.6, *p* = 0.01), which did not reach Bonferroni corrected significance level (*α* = .0025). Bayesian multiple regression showed no clear evidence for this relation for AH (*BF*_*10*_ = 1.13).

*LI*_*raw*_. For AH, we found significant associations between stronger LI_raw_ in the PFC and greater CSD-based volume (*β* = 36.45, *SE* = 8.43, *t*_(47)_ = 4.32, *p* < 0.001) and HMOA (*β* = 57.16, *SE* = 17.58, *t*_(47)_ = 3.25, *p* = 0.002) of CC-I. Bayesian multiple regression showed clear evidence for this relation (*BF*_*10*_ = 4.11). Also, for AH, there were associations between stronger LI_raw_ in the PM-SMA and greater CSD-based volume (*β* = 18.51, *SE* = 7.07, *t*_(47)_ = 2.62, *p* = 0.01) and HMOA volume (*β* = 88.04, *SE* = 30.2, *t*_(47)_ = 2.91, *p* = 0.006) of CC-II. Both relations did not reach *α* = .0025 with no clear evidence (*BF*_*10*_ = 1.73). Finally, we found an association between stronger LI_raw_ in the PTOLs and greater CSD-based volume of CC-V (*β* = 6.57, *SE* = 2.98, *t*_(47)_ = 2.2, *p* = 0.03), which did not reach *α* = .0025, with no clear evidence (*BF*_*10*_ = 1.03). Detailed summaries of the results of general and Bayesian multiple regressions nested by the groups of handedness are presented in the (S6–S8 Tables).

## Discussion

The current study aimed at verifying the link between the structural properties of each callosal sub-region and the degree of language lateralization in the corresponding cortical area. Hofer’s scheme was applied to segment the CC into five functionally specialized sub-regions, whose microstructural properties are also distinct [46]. In addition to previously reported DTI-based microstructural properties, the volumes of the callosal sub-regions, for the first time, were explored in relation to the degree of language lateralization. Also, to avoid the limitations of DTI, we additionally applied the advanced CSD technique for CC modelling and directly compared the results of the two approaches. Language lateralization was measured using the sentence completion task, which activates both anterior and posterior language-related areas within the same paradigm, unlike in previous studies [41–43]. Following [54] we focused on the degree of language lateralization regardless of the hemisphere. However, we also conducted supplementary analyses given the hemisphere of language lateralization and handedness.

Structurally, for each callosal sub-region, we revealed a significantly greater volume in CSD than in DTI. This is in line with Steventon et al. [31] and emphasizes that CSD can provide fuller reconstruction of the crossing callosal fibers, which is particularly relevant for lateral fibers projecting to language-related areas. To study the microstructural properties of callosal sub-regions, we used FA in DTI and HMOA in CSD. It is suggested that HMOA mirrors changes in the axonal diameter, fiber density and dispersion across the distinct callosal sub-regions more reliably than FA [39]. Both FA and HMOA were significantly different across the callosal sub-regions, except for the comparisons in FA between CC-II and CC-IV, between CC-III and CC-V; and in HMOA between CC-III and CC-IV. Thus, using both metrics we confirmed the microstructural heterogeneity of fibers across callosal sub-regions. We found the highest FA in CC-V, which is in line with [28, 46, 62]. But, in contrast to those studies using FA within a voxel, we extracted FA in streamlines of each callosal sub-region. That led to the discrepancy in the lowest FA that was shown in CC-I in our study but was previously reported in CC-III and CC-IV [28, 46, 62]. Similarly to FA, the highest value of HMOA was in CC-V and the lowest value of HMOA was also observed in CC-I. However, the lowest values of both FA and HMOA in CC-I are consistent with Friedrich et al. [37], who showed myelin content and axonal density based on neurite orientation dispersion and density imaging (NODDI) decreasing in the frontal segments of the CC.

The variability of the microstructural properties and potential functional specialization of the callosal sub-regions led us to consider the degree of language lateralization separately in each of the cortical areas corresponding to the terminations of the callosal sub-regions. The five resulting areas were not restricted to the core language-related regions [49], but a significant effect was found only for the area that included the posterior language-related regions. This obtained positive relation in the CSD analysis points to their inhibition of the activity in the homologous areas in the subdominant hemisphere through the callosal fibers of CC-V, which is consistent with the results for the callosal fibers projecting into the temporal regions in Josse et al. [25]. However, this study also reported that the anterior language-related areas inhibit activity of the homologous areas through the corresponding callosal fibers, whereas, in our study, this effect was not significant after multiple testing corrections. Based on Bayesian statistics of no clear evidence in favor of either a significant or null effect, we did not reject a relation between greater CSD-based volumes of CC-II and a stronger degree of language lateralization in the PM-SMA and, thus, pointed to a need to verify this relation in further studies.

The link between the volume of CC-V and the degree of language lateralization might be explained by the role of the posterior callosal sub-region in language comprehension [63]. Patients with complete or partial agenesis of CC performed a language comprehension task with lower scores than healthy participants. Moreover, for these patients, weaker language lateralization and interhemispheric connectivity were more common [64]. Although both these findings were considered within the excitation through the CC, we assume that the inhibitory model might also explain it by associating weaker language lateralization with reduced interhemispheric connectivity. Hinkley et al. [16] and Ocklenburg, Ball, Wolf, Genç, and Güntürkün [17], using auditory and dichotic listening tasks, respectively, also found a weaker degree of language lateralization in such patients, thus, confirming our result. By contrast, in healthy participants, Westerhausen et al. [22] and Bartha-Doering et al. [24] confirmed excitation of both hemispheres through the auditory callosal projections and the posterior callosal sub-region, respectively. However, those studies were limited by using the midsagittal size to represent the CC volume. Notably, the midsagittal size was used as a proxy for the volume of callosal fibers in most previous studies, whereas the present study was the first to apply tractography techniques. The fact that this effect was only found in the CSD analysis again emphasizes the advantage of CSD over DTI to feed more anatomically adequate white matter reconstructions that correspond to functional brain metrics. To our knowledge, there is only one CSD study that supports the opposite–an excitation through of the CC for the degree of language lateralization [54]. However, we suggest that our study has methodological advantages and thus provides more reliable results. First, in [54] the authors calculated the degree of functional asymmetry based on several cognitive functions, which is not specific [65]. Furthermore, their measurement of the whole body of the CC eliminated potential specific associations between each callosal sub-region and the degree of language lateralization in the corresponding cortical areas [16]. Following the idea in [10], we separated the CC into sub-regions and showed a specific association of the volume and degree of language lateralization in the area that contained the posterior language-related regions (angular and supramarginal gyri, posterior temporal cortex). Respectively, we found no link between the volume of the CC and degree of language lateralization in the PFC, in M1, nor in S1, which are not specialized for language processing but are rather involved in executive control, primary motor, and somatosensory processing, respectively [51–53].

Previous studies on language reorganization revealed that the relation of the homologous areas through the callosal subregions is not limited to inhibition. Regarding the excitatory model, greater callosal connectivity based on FA was associated with decreased language lateralization in patients with brain tumors [66] and arteriovenous malformations [67]. Despite this conflict in the results, all together seem to be explained by the switching of inhibition or excitation according to the state of the brain. Because the dominant hemisphere affected by pathology tends to recruit other regions in patients, it is believed that excitation through the callosal fibers is more efficient in performing language tasks than inhibition, which is the opposite implemented under a healthy state [68]. But a shift to the sub-dominant hemisphere as language reorganization was shown to be ineffective for better recovery in post-stroke patients and appeared to be more frequent for early strokes [69]. As a result, we can assume that the inhibitory model is regular for language tasks under a healthy or close-to-healthy state, which is in line with the results for the callosal fibers of the CC-V in our study.

Additionally, according to our results, there was no significant relation between the degree of language lateralization and either FA (in DTI) or HMOA (in CSD) of the callosal sub-regions. We found a positive association between the degree of language lateralization in the PTOLs and FA in CC-V, which did not survive a multiple testing correction. Thus, we failed to replicate the findings of Häberling et al. [28] and Steinmann et al. [27], who showed a negative link between FA in the CC and whole-brain lateralization for language and, in more detail, between FA of the posterior callosal sub-region and language lateralization in the secondary auditory cortex. One may consider that DTI-based FA indirectly represents the white-matter microstructural properties [37], but the analysis of the CSD-based HMOA of the callosal sub-regions was not associated with the degree of language lateralization either. This is seemingly in contrast to the results of Chechlacz et al. [40], who used HMOA to show the inhibition through the posterior parietal callosal fibers in enabling lateralization of spatial attention. However, this discrepancy may be due to different contributions of the callosal fibers in two different tasks, namely language and spatial attention [19, 20]. Thus, overall, the microstructural properties of the callosal fibers did not yield any effect in relation to the degree of language lateralization in our study, irrespective of the tractography approach.

While we specifically explored the absolute values of the degree of language lateralization regardless of the hemisphere [54], we additionally verified it regarding the hemispheric dominance, that is, considered both negative and positive values. In such a case, values closer to -1 represent weaker language lateralization compared to values of 0. It means that both models implemented through the CC, excitation and inhibition, occur as the functional activation and suppression of the right hemisphere by the left, dominant hemisphere, but never *vice versa*. Based on this view, surprisingly, we observed a link of greater CSD-based volumes and HMOA of CC-I to a stronger language lateralization in the PFC for left-handed with ambidextrous participants. However, there were no such associations for the callosal sub-regions terminating in the areas with the core language-related areas for any groups of the participants. Therefore, inhibition through CC-V was shown when we ignored the hemisphere, considering the absolute values of the degree only. Furthermore, our main result was significant for all participants and not replicated within the groups divided by handedness, separately. Given that we did not initially design our study to consider the groups of handedness, further studies should address it using larger samples.

The study had several limitations. Firstly, the results of Hofer’s scheme suggest clear boundaries between the callosal sub-regions. But such clear boundaries are not anatomically likely, and non-homologous projections of the CC are reported [70]. However, at least to a certain degree, our approach seems to be an appropriate approximation. Another limitation concerns the inclusion of the occipital lobe into PTOLs along with the core language-related areas–the angular and supramarginal gyri, and the posterior temporal cortex. Thus, the degree of language lateralization and CC metrics in the PTOLs reflect both high- and low-order language-related processes, despite the possibility that the link between the structural properties of the callosal sub-region and the degree of language lateralization in the high- and low-order aspects might be different. But the inclusion of the occipital lobe was motivated by the highly overlapping temporal, parietal, and occipital callosal fibers of CC-V [46], which would make it difficult to separate the occipital callosal fibers from the other fibers of CC-V. Overall, all these limitations should be addressed in further studies.

## Conclusion

In conclusion, this is the first tractography study that investigated the relation between the volumes and microstructural properties of callosal sub-regions and the degree of language lateralization, using both DTI and CSD. We showed no effect of the microstructural properties of callosal fibers on the degree of language lateralization, irrespective of the tractography method. In line with the inhibitory model, greater volumes in CSD, although not in DTI, predicted a stronger degree of language lateralization in the area containing posterior language-related regions–posterior parietal/temporal/occipital lobes. Thus, the influence of callosal fibers on the degree of language lateralization is not equipotential, but rather anatomically specific. Also, CSD was confirmed to be a more appropriate tractography approach, when lateral crossing projections are in focus, as is the case in language studies.

## Supporting information

S1 Table Descriptive statistics of the LI_raw_ and LI_abs_ in the cortical areas according to handedness of the participants. (DOCX)

S2 Table Descriptive statistics of the callosal metrics according to handedness of the participants. (DOCX)

S3 Table Results of Bayesian multiple regressions in DTI and CSD examining the relations of LI_abs_ to volumes and FA in DTI, and to volumes and HMOA in CSD. (DOCX)

S4 Table Results of the general multiple regressions in DTI and CSD examining the relations of LI_raw_ to volumes and FA in DTI, and to volumes and HMOA in CSD. (DOCX)

S5 Table Results of Bayesian multiple regressions in DTI and CSD examining the relations of LI_raw_ to volumes and FA in DTI, and to volumes and HMOA in CSD. (DOCX)

S6 Table Results of the general multiple regressions in DTI and CSD examining the relations of LI_abs_ to volumes and FA in DTI, and to volumes and HMOA in CSD within handedness groups. (DOCX)

S7 Table Results of the general multiple regressions in DTI and CSD examining the relations of LI_raw_ to volumes and FA in DTI, and to volumes and HMOA in CSD within handedness groups. (DOCX)

S8 Table Results of the Bayesian multiple regressions in DTI and CSD examining the relations of LI_abs_ and LI_raw_ to volumes and FA in DTI, and to volumes and HMOA in CSD within handedness groups. (DOCX)
