# Correction: Greater volumes of a callosal sub-region terminating in posterior language-related areas predict a stronger degree of language lateralization: A tractography study

**DOI:** 10.1371/journal.pone.0315045

**Published:** 2024-12-02

**Authors:** Victor Karpychev, Tatyana Bolgina, Svetlana Malytina, Victoria Zinchenko, Vadim Ushakov, Grigory Ignatyev, Olga Dragoy

There are errors in the Funding statement. The correct Funding statement is as follows: This work was supported by the Russian Science Foundation, project No. 20-18-00-399 (development of the DTI analysis pipeline) and by the Center for Language and Brain NRU Higher School of Economics, RF Government Grant, ag. No. 075-15-2021-619 (data collection and analyses). The funders had no role in study design, data collection and analysis, decision to publish, or preparation of the manuscript.
