# Supplementary material for: Greater volumes of a callosal sub-region terminating in posterior language-related areas predict a stronger degree of language lateralization: A tractography study
Source: PLoS One. 2022 Dec 15;17(12):e0276721. doi: 10.1371/journal.pone.0276721 (PMC9754228; doi:10.1371/journal.pone.0276721)
Supplement: S1 Table — (DOCX) [file pone.0276721.s001.docx]

**S1 Table. Descriptive statistics of the LI_raw_ and LI_abs_ in the cortical areas according to handedness of the participants.**

|  | **PFC** | | **PM-SMA** | | **M1** | | **S1** | | **PTOLs** | |
| --- | --- | --- | --- | --- | --- | --- | --- | --- | --- | --- |
|  | **LI_raw_** | **LI_abs_** | **LI_raw_** | **LI_abs_** | **LI_raw_** | **LI_abs_** | **LI_raw_** | **LI_abs_** | **LI_raw_** | **LI_abs_** |
| **AH** |  |  |  |  |  |  |  |  |  |  |
| *M* | 0.21 | 0.48 | 0.31 | 0.62 | 0.08 | 0.34 | 0.02 | 0.32 | 0.23 | 0.34 |
| *SD* | 0.50 | 0.25 | 0.63 | 0.31 | 0.46 | 0.32 | 0.43 | 0.27 | 0.34 | 0.23 |
| Min | -0.99 | 0.08 | -0.98 | 0.01 | -0.99 | 0 | -0.89 | 0.01 | -0.48 | 0 |
| Max | 0.85 | 0.99 | 0.99 | 0.99 | 1.0 | 1.0 | 0.98 | 0.98 | 0.73 | 0.73 |
| **TH** |  |  |  |  |  |  |  |  |  |  |
| *M* | 0.40 | 0.46 | 0.56 | 0.66 | 0.18 | 0.34 | -0.03 | 0.35 | 0.39 | 0.42 |
| *SD* | 0.30 | 0.20 | 0.41 | 0.19 | 0.40 | 0.27 | 0.44 | 0.24 | 0.24 | 0.18 |
| Min | -0.57 | 0.06 | -0.99 | 0.20 | -0.52 | 0 | -0.85 | 0.04 | -0.22 | 0.08 |
| Max | 0.99 | 0.83 | 0.93 | 0.99 | 0.83 | 0.83 | 0.83 | 0.85 | 0.84 | 0.84 |

We distinguished two groups of the participants according to their handedness, which corresponded to *typical handedness (TH)* and *atypical handedness (AH)*. TH consisted of right-handers, and AH consisted of left-handers and ambidexters. Two-sample *t-*tests (Bonferroni correction, *α* = .05/10 = .005) revealed no difference in LI_raw_ and LI_abs_ between AH and TH across all selected ROIs. PFC = prefrontal cortex; PM-SMA = premotor cortex and supplementary motor area; M1 = primary motor cortex; S1 = primary somatosensory cortex; PTOLs = parietal, temporal, and occipital lobes; M = mean; SD = standard deviation.
