# Supplementary material for: Greater volumes of a callosal sub-region terminating in posterior language-related areas predict a stronger degree of language lateralization: A tractography study
Source: PLoS One. 2022 Dec 15;17(12):e0276721. doi: 10.1371/journal.pone.0276721 (PMC9754228; doi:10.1371/journal.pone.0276721)
Supplement: S2 Table — (DOCX) [file pone.0276721.s002.docx]

**S2 Table. Descriptive statistics of the callosal metrics according to handedness of the participants.**

| **Callosal**  **metrics** | | **CC-I** | | **CC-II** | | **CC-III** | | **CC-IV** | | **CC-V** | |
| --- | --- | --- | --- | --- | --- | --- | --- | --- | --- | --- | --- |
|  |  | **AH** | **TH** | **AH** | **TH** | **AH** | **TH** | **AH** | **TH** | **AH** | **TH** |
| Volume  in DTI | *M* | 16.2 | 15.9 | 19.2 | 18.1 | 10.3 | 9.3 | 8.2 | 8.9 | 34.4 | 36.6 |
|  | *SD* | 3.1 | 2.2 | 3.0 | 4.0 | 2.3 | 2.0 | 2.2 | 1.6 | 7.3 | 4.4 |
| FA | *M* | 54.2 | 55.1 | 58.7 | 58.7 | 60.3 | 60.8 | 58.0 | 58.7 | 60.8 | 61.2 |
|  | *SD* | 2.3 | 2.1 | 1.6 | 1.9 | 1.9 | 1.9 | 1.8 | 1.7 | 1.8 | 1.6 |
| Volume in CSD | *M* | 34.7 | 32.2 | 46.1 | 42.0 | 18.2 | 17.7 | 15.0 | 14.4 | 68.0 | 67.4 |
|  | *SD* | 8.5 | 10.3 | 13.1 | 12.7 | 6.4 | 5.1 | 7.5 | 6.6 | 1.9 | 2.2 |
| HMOA | *M* | 22.3 | 22.5 | 29.7 | 29.6 | 27.4 | 25.7 | 28.3 | 26.3 | 34.9 | 35.6 |
|  | *SD* | 4.0 | 3.4 | 3.1 | 3.5 | 3.5 | 4.0 | 4.0 | 3.7 | 3.4 | 2.8 |

Both volumes in DTI and CSD are multiplied by 10^-3^; FA − 10^-2^; HMOA − 10^-3^. We distinguished two groups of the participants according to their handedness, which corresponded to *typical handedness (TH)* and *atypical handedness (AH)*. TH consisted of right-handers, and AH consisted of left-handers and ambidexters. Two-sample *t-*tests (Bonferroni correction, *α* = .05/20 = .0025) revealed no difference in both volumes in DTI and CSD, FA, and HMOA between AH and TH across all callosal sub-regions. CC = corpus callosum; FA = fractional anisotropy; DTI = diffusion tensor imaging; HMOA = hindrance modulated orientational anisotropy; CSD = constrained spherical deconvolution; M = mean; SD = standard deviation.
