# Supplementary material for: Greater volumes of a callosal sub-region terminating in posterior language-related areas predict a stronger degree of language lateralization: A tractography study
Source: PLoS One. 2022 Dec 15;17(12):e0276721. doi: 10.1371/journal.pone.0276721 (PMC9754228; doi:10.1371/journal.pone.0276721)
Supplement: S3 Table — (DOCX) [file pone.0276721.s003.docx]

**S3 Table. Results of Bayesian multiple regressions in DTI and CSD examining the relations of LI_abs_ to volumes and FA in DTI, and to volumes and HMOA in CSD.**

|  | **Model on the DTI data** | | |  | **Model on the CSD data** | | |
| --- | --- | --- | --- | --- | --- | --- | --- |
|  | ***P(M)*** | ***P(M\|data)*** | ***BF_10_*** |  | ***P(M)*** | ***P(M\|data)*** | ***BF_10_*** |
| **CC-I** |  |  |  | **CC-I** |  |  |  |
| Volume | 0.25 | 0.24 | 0.88 | Volume | 0.25 | 0.29 | 1.11 |
| FA | 0.25 | 0.25 | 0.85 | HMOA | 0.25 | 0.21 | 0.83 |
| Volume + FA | 0.25 | 0.23 | 0.80 | Volume + HMOA | 0.25 | 0.24 | 0.93 |
| **CC-II** |  |  |  | **CC-II** |  |  |  |
| Volume | 0.25 | 0.27 | 0.96 | Volume | 0.25 | 0.37 | 2.12 |
| FA | 0.25 | 0.23 | 0.83 | HMOA | 0.25 | 0.16 | 0.91 |
| Volume + FA | 0.25 | 0.23 | 0.84 | Volume + HMOA | 0.25 | 0.29 | 1.67 |
| **CC-III** |  |  |  | **CC-III** |  |  |  |
| Volume | 0.25 | 0.25 | 0.84 | Volume | 0.25 | 0.23 | 0.85 |
| FA | 0.25 | 0.24 | 0.83 | HMOA | 0.25 | 0.27 | 0.98 |
| Volume + FA | 0.25 | 0.22 | 0.75 | Volume + HMOA | 0.25 | 0.23 | 0.85 |
| **CC-IV** |  |  |  | **CC-IV** |  |  |  |
| Volume | 0.25 | 0.25 | 0.89 | Volume | 0.25 | 0.25 | 0.89 |
| FA | 0.25 | 0.24 | 0.83 | HMOA | 0.25 | 0.24 | 0.84 |
| Volume + FA | 0.25 | 0.23 | 0.79 | Volume + HMOA | 0.25 | 0.22 | 0.78 |
| **CC-V** |  |  |  | **CC-V** |  |  |  |
| Volume | 0.25 | 0.22 | 1.12 | Volume | 0.25 | 0.56 | 4.00^a^ |
| FA | 0.25 | 0.29 | 1.46 | HMOA | 0.25 | 0.06 | 0.83 |
| Volume + FA | 0.25 | 0.28 | 1.41 | Volume + HMOA | 0.25 | 0.29 | 2.37 |

Model on the DTI data: a model with both volume and FA, two models with either volume or FA. Model on the CSD data: a model with both volume and HMOA, two models with either volume or HMOA. *P(M)* = a prior probability of models; *P(M|data)* = posterior probability of models; BF_10_ = Bayes factor; CC = corpus callosum; FA = fractional anisotropy; DTI = diffusion-tensor imaging; HMOA = hindrance modulated orientational anisotropy; CSD = constrained spherical deconvolution.

^a^ Models with evidence for a relation at *BF_10_* > 3; scale value, *σ* = 0.036.
