# Supplementary material for: Greater volumes of a callosal sub-region terminating in posterior language-related areas predict a stronger degree of language lateralization: A tractography study
Source: PLoS One. 2022 Dec 15;17(12):e0276721. doi: 10.1371/journal.pone.0276721 (PMC9754228; doi:10.1371/journal.pone.0276721)
Supplement: S4 Table — (DOCX) [file pone.0276721.s004.docx]

**S4 Table. Results of the general multiple regressions in DTI and CSD examining the relations of LI_raw_ to volumes and FA in DTI, and to volumes and HMOA in CSD**

|  | **Global model** | | | | **Selected model** | | | |
| --- | --- | --- | --- | --- | --- | --- | --- | --- |
|  | ***β*** | ***SE*** | ***t*(47)** | ***p*** | ***β*** | ***SE*** | ***t*(48)** | ***p*** |
| **CC-I** |  |  |  |  |  |  |  |  |
| **DTI** |  |  |  |  |  |  |  |  |
| (Intercept) | -0.89 | 1.59 | 0.79 | 0.58 | -0.95 | 1.53 | -0.62 | 0.54 |
| Volume | -3.50 | 23.15 | -0.15 | 0.88 | NA | NA | NA | NA |
| FA | 2.26 | 2.83 | 0.80 | 0.43 | 2.26 | 2.81 | 0.80 | 0.42 |
| **CSD** |  |  |  |  |  |  |  |  |
| (Intercept) | -0.95 | 0.44 | -2.2 | 0.03 | -0.31 | 0.22 | -1.42 | 0.16 |
| Volume | 19.65 | 6.33 | 3.1 | 0.003^*^ | 17.83 | 6.36 | 0.37 | 0.007 |
| HMOA | 26.1 | 15.40 | 0.23 | 0.1 | NA | NA | NA | NA |
| **CC-II** |  |  |  |  |  |  |  |  |
| **DTI** |  |  |  |  |  |  |  |  |
| (Intercept) | -1.15 | 2.72 | -0.42 | 0.67 | -0.25 | 0.44 | -0.56 | 0.58 |
| Volume | 34.0 | 23.44 | 1.45 | 0.15 | 34.98 | 23.0 | 1.52 | 0.14 |
| FA | 1.57 | 4.66 | 0.34 | 0.74 | NA | NA | NA | NA |
| **CSD** |  |  |  |  |  |  |  |  |
| (Intercept) | -1.5 | 0.76 | -1.97 | 0.05 | -0.15 | 0.28 | -0.56 | 0.58 |
| Volume | 13.64 | 5.83 | 2.31 | 0.03 | 12.7 | 5.97 | 2.13 | 0.04 |
| HMOA | 44.23 | 23.43 | 1.89 | 0.06 | NA | NA | NA | NA |
| **CC-III** |  |  |  |  |  |  |  |  |
| **DTI** |  |  |  |  |  |  |  |  |
| (Intercept) | 1.48 | 2.08 | 0.71 | 0.48 | 1.28 | 1.98 | 0.65 | 0.52 |
| Volume | -9.69 | 28.57 | -0.34 | 0.74 | NA | NA | NA | NA |
| FA | -2.08 | 3.33 | -0.63 | 0.53 | -1.92 | 3.27 | -0.59 | 0.56 |
| **CSD** |  |  |  |  |  |  |  |  |
| (Intercept) | 0.37 | 0.53 | 0.69 | 0.49 | 0.26 | 0.2 | 1.28 | 0.21 |
| Volume | -8.16 | 10.97 | -0.74 | 0.46 | -7.72 | 10.69 | -0.72 | 0.47 |
| HMOA | -3.77 | 16.97 | -0.22 | 0.82 | NA | NA | NA | NA |
| **CC-IV** |  |  |  |  |  |  |  |  |
| **DTI** |  |  |  |  |  |  |  |  |
| (Intercept) | -0.58 | 2.0 | -0.28 | 0.78 | 0.27 | 0.26 | 1.0 | 0.32 |
| Volume | -34.7 | 31.6 | -1.1 | 0.28 | -31.2 | 30.2 | -1.0 | 0.31 |
| FA | 1.5 | 3.57 | 0.42 | 0.68 | NA | NA | NA | NA |
| **CSD** |  |  |  |  |  |  |  |  |
| (Intercept) | 0.12 | 0.46 | 0.26 | 0.79 | 0.11 | 0.43 | 0.24 | 0.81 |
| Volume | -0.94 | 8.76 | -0.11 | 0.91 | NA | NA | NA | NA |
| HMOA | -3.87 | 15.74 | -0.25 | 0.81 | -3.81 | 15.57 | -0.24 | 0.81 |
| **CC-V** |  |  |  |  |  |  |  |  |
| **DTI** |  |  |  |  |  |  |  |  |
| (Intercept) | -2.28 | 1.53 | -1.48 | 0.14 | -2.35 | 1.53 | -1.54 | 0.13 |
| Volume | 6.62 | 7.1 | 0.93 | 0.35 | NA | NA | NA | NA |
| FA | 3.82 | 2.56 | 1.49 | 0.14 | 4.32 | 2.5 | 1.73 | 0.09 |
| **CSD** |  |  |  |  |  |  |  |  |
| (Intercept) | -0.4 | 0.52 | -0.78 | 0.44 | -0.07 | 0.15 | -0.5 | 0.62 |
| Volume | 5.35 | 2.16 | 2.56 | 0.01 | 5.4 | 2.14 | 2.53 | 0.01 |
| HMOA | 9.0 | 13.64 | 0.66 | 0.51 | NA | NA | NA | NA |

Global model: a model with the volume and FA in DTI; the volume and HMOA in CSD.

Selected model: a model with either volume or FA in DTI; either volume or HMOA in CSD. CC = corpus callosum; FA = fractional anisotropy; DTI = diffusion tensor imaging; HMOA = hindrance modulated orientational anisotropy; CSD = constrained spherical deconvolution; SE = standard error; NA = not applicable.

*Predictors significant at *α* = .005 Bonferroni corrected.
