# Supplementary material for: Greater volumes of a callosal sub-region terminating in posterior language-related areas predict a stronger degree of language lateralization: A tractography study
Source: PLoS One. 2022 Dec 15;17(12):e0276721. doi: 10.1371/journal.pone.0276721 (PMC9754228; doi:10.1371/journal.pone.0276721)
Supplement: S5 Table — (DOCX) [file pone.0276721.s005.docx]

**S5 Table. Results of Bayesian multiple regressions in DTI and CSD examining the relations of LI_raw_ to volumes and FA in DTI, and to volumes and HMOA in CSD.**

|  | **Model on the DTI data** | | |  | **Model on the CSD data** | | |
| --- | --- | --- | --- | --- | --- | --- | --- |
|  | ***P(M)*** | ***P(M\|data)*** | ***BF_10_*** |  | ***P(M)*** | ***P(M\|data)*** | ***BF_10_*** |
| **CC-I** |  |  |  | **CC-I** |  |  |  |
| Volume | 0.25 | 0.24 | 0.83 | Volume | 0.25 | 0.34 | 2.55 |
| FA | 0.25 | 0.25 | 0.88 | HMOA | 0.25 | 0.12 | 0.93 |
| Volume + FA | 0.25 | 0.22 | 0.78 | Volume + HMOA | 0.25 | 0.39 | 2.97 |
| **CC-II** |  |  |  | **CC-II** |  |  |  |
| Volume | 0.25 | 0.28 | 1.05 | Volume | 0.25 | 0.26 | 1.41 |
| FA | 0.25 | 0.22 | 0.85 | HMOA | 0.25 | 0.21 | 1.10 |
| Volume + FA | 0.25 | 0.24 | 0.89 | Volume + HMOA | 0.25 | 0.35 | 1.85 |
| **CC-III** |  |  |  | **CC-III** |  |  |  |
| Volume | 0.25 | 0.16 | 0.29 | Volume | 0.25 | 0.25 | 0.86 |
| FA | 0.25 | 0.68 | 0.32 | HMOA | 0.25 | 0.24 | 0.83 |
| Volume + FA | 0.25 | 0.08 | 0.13 | Volume + HMOA | 0.25 | 0.22 | 0.77 |
| **CC-IV** |  |  |  | **CC-IV** |  |  |  |
| Volume | 0.25 | 0.26 | 0.92 | Volume | 0.25 | 0.24 | 0.83 |
| FA | 0.25 | 0.23 | 0.83 | HMOA | 0.25 | 0.24 | 0.83 |
| Volume + FA | 0.25 | 0.23 | 0.81 | Volume + HMOA | 0.25 | 0.22 | 0.74 |
| **CC-V** |  |  |  | **CC-V** |  |  |  |
| Volume | 0.25 | 0.23 | 0.97 | Volume | 0.25 | 0.37 | 1.91 |
| FA | 0.25 | 0.28 | 1.14 | HMOA | 0.25 | 0.16 | 0.84 |
| Volume + FA | 0.25 | 0.25 | 1.04 | Volume + HMOA | 0.25 | 0.28 | 1.47 |

Model on the DTI data: a model with both volume and FA, two models with either volume or FA. Model on the CSD data: a model with both volume and HMOA, two models with either volume or HMOA. *P(M)* = a prior probability of models; *P(M|data)* = posterior probability of models; BF_10_ = Bayes factor; CC = corpus callosum; FA = fractional anisotropy; DTI = diffusion-tensor imaging; HMOA = hindrance modulated orientational anisotropy; CSD = constrained spherical deconvolution.
