# Supplementary material for: Greater volumes of a callosal sub-region terminating in posterior language-related areas predict a stronger degree of language lateralization: A tractography study
Source: PLoS One. 2022 Dec 15;17(12):e0276721. doi: 10.1371/journal.pone.0276721 (PMC9754228; doi:10.1371/journal.pone.0276721)
Supplement: S6 Table — (DOCX) [file pone.0276721.s006.docx]

**S6 Table. Results of the general multiple regressions in DTI and CSD examining the relations of LI_abs_ to volumes and FA in DTI, and to volumes and HMOA in CSD within handedness groups.**

|  | **Model on the DTI data** | | | |  | **Model on the CSD data** | | | |
| --- | --- | --- | --- | --- | --- | --- | --- | --- | --- |
|  | ***β*** | ***SE*** | ***t*(44)** | ***p*** |  | ***β*** | ***SE*** | ***t*(44)** | ***p*** |
| **CC-I** |  |  |  |  | **CC-I** |  |  |  |  |
| (Intercept) | -0.57 | 1.1 | -0.52 | 0.61 | (Intercept) | 0.15 | 0.36 | 0.43 | 0.67 |
| Group | 1.67 | 1.78 | 0.94 | 0.35 | Group | 0.21 | 0.52 | 0.41 | 0.68 |
| AH:Volume | -11.29 | 14.06 | -0.80 | 0.43 | AH:Volume | 6.0 | 5.4 | 1.11 | 0.27 |
| TH:Volume | 22.73 | 27.02 | 0.84 | 0.40 | TH:Volume | 6.47 | 5.17 | 1.25 | 0.22 |
| AH:FA | 2.27 | 1.9 | 1.19 | 0.24 | AH:HMOA | 5.25 | 11.27 | 0.47 | 0.64 |
| TH:FA | -1.82 | 2.79 | -0.65 | .52 | TH:HMOA | -5.21 | 15.5 | -0.34 | 0.74 |
| **CC-II** |  |  |  |  | **CC-II** |  |  |  |  |
| (Intercept) | -0.49 | 1.8 | -0.27 | 0.79 | (Intercept) | 0.39 | 0.49 | 0.79 | 0.43 |
| Group | 3.02 | 2.62 | 1.15 | 0.25 | Group | 0.45 | 0.72 | 0.62 | 0.53 |
| AH:Volume | 33.56 | 17.1 | 1.96 | 0.06 | AH:Volume | 12.24 | 3.55 | 3.44 | 0.001^*^ |
| TH:Volume | -1.46 | 15.42 | -0.09 | 0.92 | TH:Volume | 0.21 | 4.49 | 0.05 | 0.96 |
| AH:FA | 0.79 | 3.15 | 0.25 | 0.8 | AH:HMOA | -11.27 | 15.17 | -0.74 | 0.46 |
| TH:FA | -3.15 | 3.21 | -0.98 | 0.33 | TH:HMOA | -6.4 | 16.16 | -0.40 | 0.69 |
| **CC-III** |  |  |  |  | **CC-III** |  |  |  |  |
| (Intercept) | -0.79 | 1.72 | -0.46 | 0.65 | (Intercept) | 0.64 | 0.51 | 1.26 | 0.21 |
| Group | 2.7 | 2.84 | 0.95 | 0.35 | Group | 0.08 | 0.77 | 0.1 | 0.92 |
| AH:Volume | -38.52 | 23.48 | -1.64 | 0.11 | AH:Volume | 1.99 | 9.12 | 0.22 | 0.83 |
| TH:Volume | 47.69 | 33.39 | 1.42 | 0.16 | TH:Volume | 2.68 | 14.16 | 0.19 | 0.85 |
| AH:FA | 2.53 | 2.79 | 0.91 | 0.37 | AH:HMOA | -12.49 | 16.42 | -0.76 | 0.45 |
| TH:FA | -3.31 | 3.58 | -0.93 | 0.36 | TH:HMOA | -16.69 | 18.0 | -0.93 | 0.36 |
| **CC-IV** |  |  |  |  | **CC-IV** |  |  |  |  |
| (Intercept) | -0.81 | 1.61 | -0.51 | 0.62 | (Intercept) | 0.24 | 0.40 | 0.61 | 0.54 |
| Group | 4.44 | 2.63 | 1.69 | 0.09 | Group | -0.02 | 0.60 | -0.04 | 0.97 |
| AH:Volume | 23.58 | 23.36 | 1.0 | 0.32 | AH:Volume | 5.5 | 6.86 | 0.8 | 0.43 |
| TH:Volume | -25.69 | 37.73 | -0.68 | 0.5 | TH:Volume | 2.12 | 9.79 | 0.22 | 0.83 |
| AH:FA | 1.62 | 2.88 | 0.56 | 0.58 | AH:HMOA | -0.3 | 12.94 | -0.02 | 0.98 |
| TH:FA | -5.19 | 3.47 | -1.49 | 0.14 | TH:HMOA | 3.79 | 17.54 | 0.22 | 0.83 |
| **CC-V** |  |  |  |  | **CC-V** |  |  |  |  |
| (Intercept) | -1.3 | 1.31 | -0.99 | 0.33 | (Intercept) | -0.08 | 0.43 | -0.20 | 0.84 |
| Group | -1.14 | 2.29 | -0.5 | 0.62 | Group | 0.19 | 0.74 | 0.25 | 0.8 |
| AH:Volume | 4.44 | 5.47 | 0.81 | 0.42 | AH:Volume | 5.23 | 2.0 | 2.6 | 0.01 |
| TH:Volume | 11.5 | 10.86 | 1.1 | 0.29 | TH:Volume | 3.71 | 2.1 | 1.79 | 0.08 |
| AH:FA | 2.44 | 2.21 | 1.1 | 0.27 | AH:HMOA | 1.89 | 11.12 | 0.17 | 0.86 |
| TH:FA | 3.96 | 2.99 | 1.32 | 0.19 | TH:HMOA | 1.9 | 16.23 | 0.12 | 0.9 |

Model on the DTI data: a model with both volume and FA nested by the groups of handedness.

Model on the CSD data: a model with both volume and HMOA nested by the groups of handedness. CC = corpus callosum; FA = fractional anisotropy; DTI = diffusion tensor imaging; HMOA = hindrance modulated orientational anisotropy; CSD = constrained spherical deconvolution; SE = standard error; NA = not applicable.

*Predictors significant at *α* = .0025 Bonferroni corrected.
