# Supplementary material for: Greater volumes of a callosal sub-region terminating in posterior language-related areas predict a stronger degree of language lateralization: A tractography study
Source: PLoS One. 2022 Dec 15;17(12):e0276721. doi: 10.1371/journal.pone.0276721 (PMC9754228; doi:10.1371/journal.pone.0276721)
Supplement: S7 Table — (DOCX) [file pone.0276721.s007.docx]

**S7 Table. Results of the general multiple regressions in DTI and CSD examining the relations of LI_raw_ to volumes and FA in DTI, and to volumes and HMOA in CSD within handedness groups.**

|  | **Model on the DTI data** | | | |  | **Model on the CSD data** | | | |
| --- | --- | --- | --- | --- | --- | --- | --- | --- | --- |
|  | ***β*** | ***SE*** | ***t*(44)** | ***p*** |  | ***β*** | ***SE*** | ***t*(44)** | ***p*** |
| **CC-I** |  |  |  |  | **CC-I** |  |  |  |  |
| (Intercept) | -0.72 | 2.13 | -0.34 | 0.73 | (Intercept) | -2.31 | 0.56 | -4.10 | <0.001^*^ |
| Group | 1.42 | 3.45 | 0.41 | 0.68 | Group | 2.73 | 0.81 | 3.35 | 0.002^*^ |
| AH:Volume | -10.06 | 27.29 | -0.37 | 0.71 | AH:Volume | 36.45 | 8.43 | 4.32 | <0.001^*^ |
| TH:Volume | 38.15 | 52.44 | 0.73 | 0.47 | TH:Volume | 12.59 | 8.06 | 1.56 | 0.12 |
| AH:FA | 2.03 | 3.69 | 0.55 | 0.59 | AH:HMOA | 57.16 | 17.58 | 3.25 | 0.002^*^ |
| TH:FA | -1.64 | 5.41 | -0.30 | 0.76 | TH:HMOA | -18.40 | 24.19 | -0.76 | 0.45 |
| **CC-II** |  |  |  |  | **CC-II** |  |  |  |  |
| (Intercept) | -0.93 | 3.71 | -0.25 | 0.80 | (Intercept) | -3.16 | 0.99 | -3.19 | 0.002^*^ |
| Group | 0.12 | 5.39 | 0.02 | 0.98 | Group | 3.23 | 1.43 | 2.32 | 0.02 |
| AH:Volume | 66.24 | 35.15 | 1.88 | 0.06 | AH:Volume | 18.51 | 7.07 | 2.62 | 0.01 |
| TH:Volume | 20.62 | 31.70 | 0.65 | 0.52 | TH:Volume | 11.54 | 8.95 | 1.29 | 0.20 |
| AH:FA | -0.06 | 6.49 | -0.01 | 0.99 | AH:HMOA | 88.04 | 30.21 | 2.91 | 0.005 |
| TH:FA | 1.69 | 6.60 | 0.26 | 0.80 | TH:HMOA | -3.21 | 32.18 | -0.10 | 0.92 |
| **CC-III** |  |  |  |  | **CC-III** |  |  |  |  |
| (Intercept) | 0.80 | 2.60 | 0.31 | 0.76 | (Intercept) | -0.91 | 0.70 | -1.29 | 0.20 |
| Group | 1.55 | 4.30 | 0.36 | 0.72 | Group | 2.56 | 1.06 | 2.42 | 0.02 |
| AH:Volume | -36.14 | 35.54 | -1.02 | 0.31 | AH:Volume | -3.96 | 12.54 | -0.32 | 0.75 |
| TH:Volume | 55.88 | 50.55 | 1.11 | 0.27 | TH:Volume | -13.25 | 19.46 | -0.68 | 0.50 |
| AH:FA | -0.58 | 4.23 | -0.14 | 0.89 | AH:HMOA | 38.56 | 22.57 | 1.71 | 0.09 |
| TH:FA | -4.42 | 5.42 | -0.81 | 0.42 | TH:HMOA | -48.0 | 24.75 | -1.94 | 0.06 |
| **CC-IV** |  |  |  |  | **CC-IV** |  |  |  |  |
| (Intercept) | -0.48 | 2.74 | -0.17 | 0.86 | (Intercept) | -0.31 | 0.65 | -0.48 | 0.64 |
| Group | -0.44 | 4.48 | -0.10 | 0.92 | Group | 1.06 | 0.98 | 1.08 | 0.29 |
| AH:Volume | -32.66 | 39.77 | -0.82 | 0.42 | AH:Volume | 4.80 | 11.15 | 0.43 | 0.67 |
| TH:Volume | -33.38 | 64.22 | -0.52 | 0.61 | TH:Volume | -6.77 | 15.90 | -0.43 | 0.67 |
| AH:FA | 1.32 | 4.90 | 0.27 | 0.79 | AH:HMOA | 9.20 | 21.03 | 0.44 | 0.66 |
| TH:FA | 2.03 | 5.91 | 0.34 | 0.73 | TH:HMOA | -25.64 | 28.50 | -0.90 | 0.37 |
| **CC-V** |  |  |  |  | **CC-V** |  |  |  |  |
| (Intercept) | -1.06 | 1.95 | -0.54 | 0.59 | (Intercept) | -0.46 | 0.64 | -0.72 | 0.47 |
| Group | -2.40 | 3.39 | -0.71 | 0.48 | Group | 0.29 | 1.09 | 0.27 | 0.79 |
| AH:Volume | 4.50 | 8.09 | 0.56 | 0.58 | AH:Volume | 6.57 | 2.98 | 2.20 | 0.03 |
| TH:Volume | 11.73 | 16.07 | 0.73 | 0.47 | TH:Volume | 4.47 | 3.08 | 1.45 | 0.15 |
| AH:FA | 1.86 | 3.27 | 0.57 | 0.57 | AH:HMOA | 6.84 | 16.52 | 0.41 | 0.68 |
| TH:FA | 5.54 | 4.42 | 1.25 | 0.22 | TH:HMOA | 7.16 | 24.11 | 0.30 | 0.77 |

Model on the DTI data: a model with both volume and FA nested by the groups of handedness. Model on the CSD data: a model with botn volume and HMOA nested by the groups of handedness. CC = corpus callosum; FA = fractional anisotropy; DTI = diffusion tensor imaging; HMOA = hindrance modulated orientational anisotropy; CSD = constrained spherical deconvolution; SE = standard error; NA = not applicable.

*Predictors significant at *α* = .0025 Bonferroni corrected.
