# Supplementary material for: Greater volumes of a callosal sub-region terminating in posterior language-related areas predict a stronger degree of language lateralization: A tractography study
Source: PLoS One. 2022 Dec 15;17(12):e0276721. doi: 10.1371/journal.pone.0276721 (PMC9754228; doi:10.1371/journal.pone.0276721)
Supplement: S8 Table — (DOCX) [file pone.0276721.s008.docx]

**S8 Table. Results of the Bayesian multiple regressions in DTI and CSD examining the relations of LI_abs_ and LI_raw_ to volumes and FA in DTI, and to volumes and HMOA in CSD within handedness groups.**

|  | **Model on the DTI data** | | |  | **Model on the CSD data** | | |
| --- | --- | --- | --- | --- | --- | --- | --- |
|  | ***P(M)*** | ***P(M\|data)*** | ***BF_10_*** |  | ***P(M)*** | ***P(M\|data)*** | ***BF_10_*** |
| **LI_abs_** | | | | | | | |
| **CC-I** |  |  |  | **CC-I** |  |  |  |
| AH | 0.25 | 0.24 | 0.96 | AH | 0.25 | 0.24 | 0.92 |
| TH | 0.25 | 0.24 | 0.93 | TH | 0.25 | 0.25 | 0.96 |
| **CC-II** |  |  |  | **CC-II** |  |  |  |
| AH | 0.25 | 0.25 | 1.0 | AH | 0.25 | 0.28 | 1.62 |
| TH | 0.25 | 0.24 | 0.95 | TH | 0.25 | 0.24 | 0.91 |
| **CC-III** |  |  |  | **CC-III** |  |  |  |
| AH | 0.25 | 0.25 | 1.0 | AH | 0.25 | 0.24 | 0.90 |
| TH | 0.25 | 0.25 | 1.0 | TH | 0.25 | 0.24 | 0.94 |
| **CC-IV** |  |  |  | **CC-IV** |  |  |  |
| AH | 0.25 | 0.24 | 0.94 | AH | 0.25 | 0.24 | 0.90 |
| TH | 0.25 | 0.25 | 0.99 | TH | 0.25 | 0.24 | 0.91 |
| **CC-V** |  |  |  | **CC-V** |  |  |  |
| AH | 0.25 | 0.24 | 0.95 | AH | 0.25 | 0.26 | 1.13 |
| TH | 0.25 | 0.25 | 1.03 | TH | 0.25 | 0.25 | 1.03 |
| **LI_raw_** | | | | | | | |
| **CC-I** |  |  |  | **CC-I** |  |  |  |
| AH | 0.25 | 0.24 | 0.90 | AH | 0.25 | 0.54 | 4.11^a^ |
| TH | 0.25 | 0.24 | 0.93 | TH | 0.25 | 0.26 | 1.04 |
| **CC-II** |  |  |  | **CC-II** |  |  |  |
| AH | 0.25 | 0.25 | 0.98 | AH | 0.25 | 0.34 | 1.73 |
| TH | 0.25 | 0.24 | 0.93 | TH | 0.25 | 0.25 | 0.98 |
| **CC-III** |  |  |  | **CC-III** |  |  |  |
| AH | 0.25 | 0.24 | 0.91 | AH | 0.25 | 0.25 | 0.98 |
| TH | 0.25 | 0.25 | 0.99 | TH | 0.25 | 0.26 | 1.06 |
| **CC-IV** |  |  |  | **CC-IV** |  |  |  |
| AH | 0.25 | 0.24 | 0.91 | AH | 0.25 | 0.24 | 0.89 |
| TH | 0.25 | 0.24 | 0.94 | TH | 0.25 | 0.24 | 0.91 |
| **CC-V** |  |  |  | **CC-V** |  |  |  |
| AH | 0.25 | 0.24 | 0.90 | AH | 0.25 | 0.25 | 1.03 |
| TH | 0.25 | 0.25 | 1.02 | TH | 0.25 | 0.25 | 1.0 |

Model on the DTI data: a model with both volume and FA nested by the groups of handedness.

Model on the CSD data: a model with both volume and HMOA nested by the groups of handedness. *P(M)* = a prior probability of models; *P(M|data)* = posterior probability of models; BF_10_ = Bayes factor; AH = atypical handedness; TH = typical handedness; CC = corpus callosum; DTI = diffusion-tensor imaging; HMOA = hindrance modulated orientational anisotropy; CSD = constrained spherical deconvolution.

^a^ Models with evidence for a relation at *BF_10_* > 3; scale value, *σ* = 0.018.
